# Facilitators and barriers for using outdoor areas in the primary work tasks of eldercare workers in nursing homes

**DOI:** 10.1186/s12913-023-10308-x

**Published:** 2023-11-24

**Authors:** Sandra Schade Jacobsen, Maja Vilhelmsen, Lene Lottrup, Mikkel Brandt

**Affiliations:** 1 National Research Centre for the Working Environment, Lersø Parkalle 105, Copenhagen, Denmark; 2 Center for Clinical Research and Prevention, Frederiksberg, Denmark; 3 Outdoor Institute, Silkeborg, Denmark; 4 Sundhedslandskab, Aarhus, Denmark

**Keywords:** Health Care professionals, Mental Health, Occupational Health, Nature, Caregivers, Health care workers, Occupational Health nursing, Goldilocks work principle

## Abstract

**Background:**

Eldercare workers in nursing homes report high musculoskeletal disorders, stressful work, and sickness absence. Initiatives that can accommodate these issues are needed. Current studies point out that nature contact may offer a range of human health benefits, potentially promoting healthier work among eldercare workers. Therefore, this study aimed to investigate facilitators and barriers for using outdoor areas as part of the daily work among eldercare workers in Danish nursing homes.

**Methods:**

In this multiple case study, we collected data from three nursing homes, conducting three semi-structured focus group interviews with eldercare workers and three individual interviews with nursing home managers. Furthermore, we conducted observations of the daily work and mappings of the nursing homes’ outdoor environments to gain in-depth knowledge of eldercare workers’ and managers’ perspectives on using outdoor areas in their daily work. The data was thematically analysed using ‘The Behaviour Change Wheel’ (BCW), more specifically the COM-B model, as a theoretical foundation for exploring facilitators and barriers for the use of outdoor areas.

**Results:**

Frequently mentioned facilitators were facilities, traditions or repetitive events, positive experiences with residents (‘star moments’), and knowledge about the residents. Frequently mentioned barriers were insufficient staffing, hierarchy in the work tasks, professional identity, and lack of ideas.

**Conclusions:**

The identified facilitators and barriers should be considered when designing initiatives for increased use of outdoor areas or activities of eldercare workers.

**Trial registration:**

According to the Danish ethics committee (Law of committee, (komitéloven) paragraph 14, Sect. 2), qualitative interviews, which do not include human biological materials, do not need neither approval by ethical and scientific committee or informed consent (The Danish National Centre for Ethics).

**Supplementary Information:**

The online version contains supplementary material available at 10.1186/s12913-023-10308-x.

## Introduction

Eldercare workers in nursing homes have physically and emotionally demanding work tasks. Work-related musculoskeletal disorders and stressful work are frequent in this occupation [1–3]. According to the Danish Work Environment & Health investigation, 46.2% of eldercare workers report having pain in their body several times per week, and 9.1% report that they are limited in their work because of pain. Furthermore, 66.9% of the eldercare workers experience work-related stress [4]. The socio-economic cost due to sickness absence and hence coming loss of productivity is dramatically high [5–7], which have huge consequences for the healthcare sector and society [8]. This situation in the health care sector is worsened by a worker shortage estimated to increase in the coming years [7, 9, 10]. Therefore, there is a need for initiatives that can reduce sickness absence, maintain eldercare workers in their jobs, and recruit more workers to the eldercare sector.

Nature contact may offer a range of human health benefits, which has the potential to promote healthier work among eldercare workers [11–14]. Studies point to a number of benefits, which include positive physical and psychological outcomes such as enhanced immune system and improved respiratory, cardiovascular, and metabolic function [15–17], as well as improved mood, attention restoration, and decrease in anxiety and depressive symptoms [14, 18–20]. Most studies focus on using outdoor areas in the context of urban development, residential areas, hospitals, or institutions, or in connection with specific health services. However, in recent years, research has also focused on the importance of companies’ outdoor areas for employees’ health and wellbeing. A Swedish study showed a number of positive consequences of working outdoors, including increased general wellbeing, balancing stress symptoms, improved communication and social relationships, increased concentration, and a sense of self-determination. However, participants also experienced guilt from being outdoors and that it was not ‘real work’ [21]. An American cross-sectional study on office workers based on questionnaires showed a significant, negative association between nature contact and stress and nature contact and general health complaints [22]. Moreover, a randomized controlled study showed that an ‘outdoor booster break’ reduced stress significantly compared to the control group that did 10-minute breaks indoors [23], and even the possibility of having a nature view and workplace greenery seems to reduce the mental stress for a variety of occupations [24–26]. The results from these studies are supported by other research showing benefits from working outdoors in terms of employees’ health, wellbeing, and job satisfaction, but also a number of physical, organizational, and cultural obstacles for moving work activities outdoors [23, 24, 26, 27].

The majority of research done within this topic has been in white-collar jobs, which are mainly characterized as sedentary and performed in the same location. The usage and possible effects of using outdoor areas within more dynamic job types, such as the eldercare sector, are not well investigated. If outdoor space can be integrated in the daily work of eldercare workers, it indicates a potential for improving physical and mental health. To enable this, facilitators and barriers for such an integration needs to be explored.

Therefore, we aimed to investigate facilitators and barriers for using outdoor areas as part of the daily work among eldercare workers in nursing homes in Denmark.

## Methods

### Design and philosophical and theoretical foundations

This field study is a multiple case study using semi-structured interviews, observations, and mapping of outdoor environments in nursing homes to gain in-depth knowledge on eldercare workers’ use of outdoor areas in their daily work, primarily focusing on facilitators and barriers. A multiple case study allows us to investigate our research objective in a real-life setting of eldercare workers, and the inclusion of three cases enables the generation of broad and nuanced knowledge about the subject [28].

According to the Danish ethics committee (law of committee, (komitéloven) paragraph 14, Sect. 2), qualitative interviews, which do not include human biological materials, do not need neither approval by ethical and scientific committee or informed consent (The Danish National Centre for Ethics). Participants consented verbally to the recording of interviews, and all data were processed and analyzed anonymously. All methods were carried out in accordance with relevant guidelines and regulations.

#### Philosophical foundation

We took a critical realist approach, as we wished to investigate and identify relationships and non-relationships between what we experience (at the nursing homes), what actually happens, and the underlying mechanisms that produce the (outdoor-related) events [29]. As critical realists, we acknowledge that our findings are influenced by our theoretical understanding and previous experiences. Furthermore, we are aware that we are not able to grasp everything empirical at the three nursing homes. Hence, our findings are not a value-free replication of reality but a social product connected to our consciousness and knowledge and the specific context at the three nursing homes.

#### Theoretical foundation for describing facilitators and barriers for use of outdoor areas

We used ‘The Behaviour Change Wheel’ (BCW) as a theoretical foundation for exploring facilitators and barriers for use of outdoor areas. The behaviour-change theory can help identify barriers to change prior to the tailoring of interventions [30], e.g., BCW has been used to explain physical activity behaviours [31], also related to work [32]. At the centre of the BCW lies the COM-B model (Capability, Opportunity, Motivation—Behaviour) (Table 1). According to the COM-B model, behaviour results from an interaction between three components: capability, opportunity, and motivation [33, 34]. Capability can be psychological (knowledge) or physical (skills), opportunity can be social (cultural norms) or physical (environment), and motivation can be automatic (emotions) or reflective (beliefs and identity). The model places no priority on one component, instead, it provides “*a way of identifying how far changing particular components or combinations of components could effect the required transformation*” of behaviour in a specific context [34].

**Table 1:** The COM-B model (Capability, Opportunity, Motivation—Behaviour model). Components, subcomponents and definitions of the COM-B model including the research group’s adapted definitions, which we created to achieve a common understanding of the coding strategy (described in the data analysis section)

COM-B component	COM-B subcomponent	COM-B definition	COM-B adapted definition
Capability	Psychological capability	Knowledge, memory, attention, decision processes, behavioural regulation.	Knowledge (e.g. about suitable walking routes in the local area or about planting flowers/greenery) and self-confidence (e.g. being able to get ideas for activities and initiate them).
	Physical capability	Skills, abilities or proficiencies acquired through practice.	Skills (e.g. the ability to make a bonfire) and physical capability (e.g. being able perform certain physical activities).
Opportunity	Physical opportunity	Environmental context and resources.	Facilities (e.g. exits, gardens, ground and surrounding area), resources (e.g. staffing and finances), and weather (e.g. snow/icy roads).
	Social opportunity	Social influences such as social pressure, norms, conformity, social comparisons.	Culture and social rules (e.g. whether employees only go outside when the weather is good, acceptance of taking breaks outside and being available to residents and colleagues).
Motivation	Reflective motivation	Beliefs about capabilities, consequences, roles, identity, intentions, goals, optimism.	Beliefs and professional identity.
	Automatic motivation	Emotions, reinforcement such as rewards incentives, punishment.	Habits and routines (e.g. “we usually do…”), impulses (e.g. “I wanted fresh air, so I went out”), and smoking breaks.

As our study is designed as a multiple case study, it is situated in three specific contexts (nursing homes) and focused on fixed aspects related to the use of outdoor areas. Thus, we decided to adapt and operationalize the definitions of the three components of COM-B to align with these aspects (Table 1). This adjustment was carried out after data collection and was based upon the preliminary analysis of the gathered data. The operationalized definitions served the purpose of establishing a shared understanding of the coding approach employed in the analysis of interviews and observations, as outlined in the data analysis section.

### Recruitment of nursing homes

A total of twenty public nursing homes located in the mid-western region of Jutland (population of 1.2 million citizens), Denmark, were invited to take part in the study. In short, we informed the nursing home managers about the study orally at a joint meeting in February 2022. Our first intention was to recruit two ‘regular’ nursing homes with no or limited use of outdoor areas and two nursing homes with an outdoor profile and focus on using outdoor areas. However, the invited nursing homes all expressed that they had very limited use of outdoor areas, and we decided to include all interested nursing homes in the municipality without criteria for size, number of employees, or specific profiles.

Five nursing homes wished to participate in the study. Due to the Covid-19 virus and the associated lack of staffing, two nursing homes withdrew from participation. Thus, three nursing homes were included in the study. One was exclusively a nursing home for residents with dementia with 18 residencies, and the two remaining nursing homes had both a somatic unit with 30–39 residencies and a smaller unit for residents with dementia with 7–10 residencies. All nursing homes had somewhat similar outdoor areas, including smaller gardens (both fenced and open), terraces, and lawns. However, the nursing homes varied in their nearby surroundings, such as accessibility to nearby parks and greenery. A total of eleven female eldercare workers, including assistants (4–24 years of seniority) and helpers (1 month to 17 years of seniority), and three female managers (1–2 years of management seniority) participated in the interviews.

### Data collection

We collected the data in March 2022 by visiting each recruited nursing home for one to two days, carrying out (1) observations of the daily work, (2) a focus group interview with 3–5 employees, (3) an individual interview with the nursing home manager, and (4) mapping of outdoor areas. The researchers conducting the data collection (SSJ, MV, and MB) are educated and experienced in collecting data at workplaces, taking observational notes, and interviewing employees.

#### Observations

We conducted overt, non-participant observations of the daily work at each nursing home. The observations were conducted by one or two researchers, who moved freely around the nursing home for approximately one workday (~ 5–7 h) while simultaneously taking notes. We kept away from enclosed dementia units and the resident’s private apartments. Inspired by BCW-framework [34], we directed a focus towards opportunities, capabilities, and motivation for the use of outdoor areas in daily work. We paid attention to social and physical opportunities (e.g., social influence and environmental context and resources) at the inside and outside areas of the nursing homes, physical and psychological capability (e.g., decision process, attention, knowledge, and skills), and automatic and reflective motivation (e.g., intentions, beliefs about consequences and emotions) of the eldercare workers.

#### Interviews

Before starting the data collection at the nursing homes, we (the research group) developed two thematic, semi-structured interview guides: one for the individual interview with the manager (see Additional file 1) and one for the focus group interview with employees (see Additional file 2). The interview guides were based on the BCW-framework [34]. The interview guides contained open-ended questions about the present use of outdoor areas at the nursing homes, attitudes towards the use of outdoor areas, and ideas and/or wishes for future use of outdoor areas at the nursing homes. At each nursing home, we conducted one individual interview with the manager and one focus group interview with a group of employees. The employees that participated in the focus group interviews were selected by the manager and depended on the work schedule. All interviews took place at the nursing home in an undisturbed room with the presence of one or two researchers, with one researcher being the primary interviewer. The interview guides supported the interviewer but were not followed strictly, giving the participants the opportunity to speak freely about their perspectives. All interviews were conducted in Danish, audio-recorded, and transcribed in Danish. Translation of quotes and observational notes were completed in the process of drafting this paper.

#### Mapping of outdoor environments

Environmental psychology describes how a certain environmental structure is suited to certain behaviour patterns [35]. In order to investigate how the location and design of the nursing homes’ outdoor areas promote or hinder employees’ use of the outdoor areas, we mapped the outdoor areas of each nursing home by photographing each area and taking descriptive notes on access, appearance, and facilities. We mapped both areas belonging to the nursing home and nearby public outdoor areas.

### Data analysis

We transcribed interviews using an intelligent verbatim approach, following a transcript protocol [36]. Transcripts and observation notes were anonymized and imported into NVivo (V.12 pro). We used an abductive thematic analysis to identify facilitators and barriers for the use of outdoor areas among nursing home workers and managers [37]. Initially, two researchers (MV and SSJ) read the transcripts and observation notes to get familiar with the data material while taking notes about facilitators and barriers. These notes were afterwards discussed and linked to the COM-B model to develop an initial coding strategy. We classified factors that positively affected going outdoors as facilitators and factors that negatively affected going outdoors as barriers. In an iterative process, MV and SSJ tested the coding strategy on two transcripts, discussed it in the research group, and refined it. To get a common understanding of the coding strategy, the research group made adapted definitions of each COM-B subcomponent (Table 1). We organized related codes into themes and sub-themes and mapped them to the constructs (capabilities, opportunities, and motivation) of the BCW-framework, using them as overarching themes. We did this to achieve a coherent and theoretically founded understanding of facilitators and barriers from an early start. In this process, we found that some themes could be labelled as both a facilitator and barriers, depending on social characteristics or contextual circumstances. Afterwards, MV, SSJ, and MB used the coding strategy to code all transcripts and observation notes and to review the mapping of outdoor areas. To secure internal homogeneity (coherence with coded data extracts) and external heterogeneity (a clear distinctions between themes and sub-themes), we reviewed, refined and renamed themes.

## Results

Utilizing the COM-B model as a theoretical foundation, we conducted six interviews with eldercare workers and managers, along with detailed observational notes and mappings of the outdoor environment at three Danish nursing homes. This approach enabled us to identify the facilitators and barriers associated with incorporating outdoor areas into daily practices. From this data, we identified facilitators and barriers for using outdoor areas in daily work routines among eldercare workers based on the overall themes; physical opportunities, social opportunities, reflective motivation, automatic motivation, psychological capability and physical capabilities. We found that factors associated with physical and social opportunities were the most prominent, while we did not find any factors related to physical capabilities. Table 2 provides an overview of the results, more specifically displaying the themes from the analysis mapped onto the subcomponents of the COM-B model.

**Table 2:** Identified facilitators and barriers for using outdoor areas as part of the daily work among eldercare workers mapped on to the subcomponents of COM-B model. Some themes from the analysis were labelled as both a facilitator and barrier, depending on social characteristics or contextual circumstances

COM-B subcomponent	Themes from the analysis		
	*Facilitator*	*Barrier*	*Both facilitator and barrier*
Physical opportunities	Facilities	Insufficient staffing	Access to outdoor areas
			Weather
Social opportunities	Traditions and repetitive events	Hierarchy in work tasks	Communication and coordination
		Being available	
Reflective motivation	‘Star moments’	Predicting consequences	-
		Professional identity	
Automatic motivation	Routines	-	-
Psychological capabilities	Knowledge about the residents	Lack of ideas	Taking responsibility
Physical capabilities	*-*	*-*	*-*

### Physical opportunities

#### Facilities

Having a variety of facilities was an important facilitating factor for doing more outdoor activities. First, the employees explained that facilities play a supportive role when being outdoors. The employees revealed that numerous residents frequently expressed concerns about the outdoors being ‘too cold’. Thus, it seems important to have facilities that can enhance the residents’ comfort, such as blankets and shelter. One employee exemplified this:

“*Being out in the sun on a chair with a couple of residents. In this weekend, we found some shelter (from the wind), where we sat down with three residents and had a nice half an hour, sitting with blankets and enjoyed it.*” Employee, case 01.

Our mapping of the outdoor environment showed that all nursing homes had patio furniture placed in different corners of their gardens. One manager touched upon this theme in an interview as well, explaining how supportive and/or technical facilities, such as extra wheelchairs, can enhance the comfort of the residents when going on longer walks:

“*We have some wheelchairs for residents who are not able to walk longer distances. For the period where the resident’s level of physical function might drop—so there will be a wheelchair to those who haven’t been granted one yet. Then you can still go for a walk.”* Manager, case 01.

Second, the employees mentioned that facilities could contribute to amusement. Employees mentioned that games such as petanque/boules or bingo facilitated specific outdoor activities, walking paths around the garden facilitated walks and vegetation often facilitated conversation. One employee exemplified this:

“*Then we (employees and residents) talk about the flowers, because we have a lot of flowers out there, and then you can have a conversation about them like ‘are the fruit trees starting to grow’.* ” Employee, case 01.

Thus, facilities seem to be a facilitator for using outdoor areas by either contributing to the residents’ comfort or amusement when going outside.

#### Weather

Facilities contributing to comfort appeared to be connected to a dual perception of the weather being defined as both a barrier and a facilitator for going outside. Employees defined it as a barrier as residents often resist going outdoors if the weather is cold, leading to the use of blankets or shelter. Further, both employees and one manager mentioned that cold and wet weather increase the risk of residents falling due to slippery ground, which might lead to the use of e.g. wheelchairs. On the contrary, ‘special weather’ such as the first snowfall or the first signs of spring, was described as a facilitator for going outdoors, as it contributed to new experiences, a change in everyday life, and a different conversation with residents. In line with this, the managers often encouraged the employees to do outdoor activities when the weather was ‘nice’ and sunny. One manager addresses this herself:

“*I often encourage them, and I think they will agree in that. Like, the weather is going to be nice today, shouldn’t you go for a walk with them (residents)?*” Manager, case 03.

In this way, the weather can either facilitate or hinder the use of outdoor areas as both management, employees, and residents perceive and act differently upon it depending on the state of it.

#### Access to outdoor areas

Employees described access to outdoor areas as both a facilitator and a barrier for going outside, depending on the characteristics of the access. Especially direct access to a ‘nice’ outdoor area, such as gardens and terraces, appeared as a key component as different departments in the same nursing home had different accesses to outdoor areas. We observed that one department had access to an enclosed garden while another department had access to a parking lot. This influenced the motivation to go outside as well as the type of activities conducted. This is exemplified in a dialogue between two colleagues during an interview (case 03):

*”You can say, in the summer, we play petanque and that kind of things at ours.”* Employee 1.

*“That is because you have the garden.”* Employee 2.

*“Because we have the garden.”* Employee 1.

*“We can’t send our residents out on the road.”* Employee 2.

Employees described advantages by having easy access to a garden. First, a garden makes it possible to go outside, but still be available to help colleagues inside. Second, nearby greenery facilitates spontaneous, short activities. In one nursing home, we observed an employee spontaneously inviting a resident outside to pick flowers in the garden. The activity only took eight minutes, but contributed to a joyful moment and conversations with three different employees, and was possible to do because of the easy garden access.

During interviews, several employees and one manager pointed out the importance of the characteristics of the outdoor areas in the local community. Our mapping of the outdoor environments revealed that the nursing homes’ surroundings varied greatly. One nursing home was located close by to a small lake with a walk-friendly path going around it, which was frequently used by the residents and employees. On the contrary, another nursing home was located on a hilltop in a residential area, which one employee mentioned as a big barrier for going on walks with the residents:

*“There are not so many places here where you can walk around with a wheelchair. It’s all hills and such, so you have to think about that too.”* Employee, case 02.

#### Insufficient staffing

Both employees and managers mentioned limited resources, more specifically insufficient staffing, as a major barrier for going outdoors. This applied to both organization of smaller spontaneous activities (e.g., a walk in the nearby area) and larger events (e.g., a full day boat trip). To feel sufficiently staffed, employees explained that they had to be able to divide into two groups, one staying inside and one going outside. This assured them in being available to the residents (also connected to Social Opportunities: Being available) and reduced the risk of feeling stressful, as described by one employee:

*“We will have to take a look at the work schedule, so how many people we are on that day. It’s no good if we are severely understaffed and have six residents each. It has to be the day where there are more hands to handle it, so that it doesn’t become stressful, both for ourselves, but also for the residents. It should be an enjoyable moment.”* Employee, case 03.

Others agreed by explaining that they had to be one-to-one with the residents when doing activities outside, almost despite the resident’s physical functional level. The managers supported this perspective. One manager explained how insufficient staffing is a barrier despite employees having some time during the workday to do outdoor activities:

*“(…) if there are only 6 people at work and there are 30 residents who needs help and personal care, etc., then there is maybe half an hour in the middle of the morning, an hour, where you could take 1–4 residents out for an hour, but you can’t manage… the two can’t manage to take more residents out.”* Manager, case 02.

According to another manager, a way to overcome the barrier of insufficient staffing could be by checking future work schedules and secure sufficient staffing on days with planned outdoor activities.

### Social opportunities

#### Traditions and repetitive events

During the interviews, when employees shared previous experiences with outdoor activities, the majority of these experiences were connected to traditions or repetitive events. Previous outdoor activities were often either something employees and residents did every year or related to a specific season or celebration, such as Easter or Christmas. One nursing home had a boat trip once a year, while another had a food truck serving Christmas cuisine outdoors in December. One manager told she was often surprised by what the employees could manage of outdoor activities when it was a tradition or a repetitive event:

“*There are the employees who will come and say, ‘this and this is a tradition, we usually bring this’ and then I think ‘wow, can they really manage that?’, but I should not become a barrier, you know. I just think like, ‘okay, they have some experience here’.*” Manager, case 01.

This indicates that traditions and repetitive events can build up experience with specific outdoor activities, making employees more comfortable in conducting them.

#### Hierarchy in work tasks

We identified that there was a clear hierarchy in work tasks, where more generic care tasks were prioritized higher than outdoor activities. This became evident both in the observations of daily practices and in the interviews. Therefore, this hierarchy was a barrier for going outdoors. When asked to describe their workday, very few employees mentioned activities outdoors. Instead, they named activities such as morning care, preparing meals, and getting the residents ready for an afternoon nap. One employee explained that there is a list of things, which need to be taken care of in the morning, and therefore, outdoor activities comes last:

”*So we are far into the morning, if we do activities, if there is time to it.*” Employee, case 03.

Our observations support this prioritization as two employees prioritized to organize tableware when given some extra time. The two employees expressed gratitude for this, and one of them mentioned that she was not in a hurry as she had ‘*until 1pm to do the activity plan’*. One manager was aware of this barrier as she during the interview highlighted her own role in communicating the prioritization of outdoor activities to employees:

“*I think that it (an outdoor activity) should come from this feeling that it is just as an important task as if you are asked to stay inside and do something more practical. So, in my opinion, it’s about trying to communicate that to the staff. That it is just as valuable that two of our demented residents (the informant’s own expression) are taken out for a half hour walk as if you clean the kitchen. It is just as much a work task as everything else, but they (the staff) do not prioritize it.*” Manager, case 02.

The quote indicates that outdoor activities is not perceived as ‘a real work task’ among employees, leading to a down-prioritizing of it in the work hierarchy.

#### Being available

During observations, we experienced a practice of wanting to be available, which was a barrier for going outside. Employees had their coffee breaks and lunch in the residents’ common area and explained that this was to be available for colleagues and residents. During an interview, one employee identified paid breaks as one of the reasons for this practice:

”*And you can’t sit there (in the office) and have your break. We sit in the common area, also to be available. We get paid during our break, so we need to be available and ready.*” Employee, case 03.

The practice seems to count besides breaks. During another interview, an employee described how she used the garden of the nursing home for shorter walks with residents to be available and within a short distance if her colleagues needed help.

It did not seem socially acceptable among employees to go for a walk alone, away from the nursing home, during breaks. When discussing this matter in one of the focus group interviews, the employees replied instantly that *“personally, I would not do that”* and *“I don’t think I would do that”.* On the contrary, the same group of employees agreed that breaks outside could have beneficial effects on their own wellbeing, as it would be an actual break from ‘being available’. One employee even explained that she sometimes felt like being *‘released from prison’* when finishing a long workday inside.

#### Communication and coordination

Across all three nursing homes, communication and coordination appeared as important factors for going outdoors. However, it could both facilitate and inhibit outdoor activities. During interviews, employees emphasized the necessity to coordinate within the team before going outdoors, e.g. in the garden. One employee explained that she would always ask her colleagues before going outdoors. Similar, in one nursing home, we observed how employees during their morning break coordinated who went for a walk with a couple of residents and who stayed back. Employees from another nursing home described a similar practice:

“*Then some will go for a walk, some will stay and do medicine dispensation, and some will take care of some documentation (…). Like today where we agree upon who will do this interview, who will stay back and who will join the music class. And it is not our manager who will organize and distribute this.*” Employee, case 03.

The quote demonstrates that communication, coordination and reaching consensus in the colleague group is important for conducting activities, e.g. going for a walk outside. One of the managers was aware of this and described in an interview how she has a responsibility in supporting coordination of activities:

“*The fewer resources (employees on job) there are, the more coordinated they (the staff) are themselves. The more people they are, the less coordinated they become. We really have to be careful how we spend our time when we are at work. Because otherwise a day slips away easily. So, I have an important task in that.*” Manager, case 01.

Thus, communication and coordination—both between employees and between manager and employees—can be an important factor having the potential to either facilitate or limit the conduction of outdoor activities.

### Reflective motivation

#### ‘Star moments’

One factor that seemed to motivate several employees was seeing the positive outcomes from having experiences outside with their residents. During an interview, one employee even described these situations as ‘star moments’. In these types of moments, conversations with the resident changes, the relationship becomes more equal, and the resident’s mood lightens. Another employee explained:

*”For me it’s about seeing a change in the resident. I can see joy, I can see… that look in their eyes the moment they go outside. Even though they might say ’uh, it’s cold’. Once you go outside and when you start talking, ’oh, look at this, the grass is slowly staring to…’. You can see the resident’s face, how it starts to change.”* Employee, case 01.

The management agreed that these moments are important for building strong connections to the residents. During an interview, one manager explained that the employee and resident are subjects to a great deal of legislation when they are inside the nursing home, which makes their relationship unequal. However, when they are outside, away from the nursing home, the relationship equalizes, and it benefits the wellbeing of both employee and resident. Additionally, one manager said that going for a walk with a resident often gives the employee a deeper knowledge about the resident:

*“I also think that sometimes they (the employees) get a lot of other knowledge when they go for a walk. Because the senses might register something (…), it may be that the resident actually opens up to something or other. So sometimes that knowledge also becomes something that must be shared in relation to the professional part.”* Manager, case 01.

In these ways, ‘star moments’, where residents lighten and opens up, can be identified as facilitator for conducting outdoor activities with residents.

#### Predicting consequences

From the interviews, we found that the employees frequently evaluated the residents’ mental and physical state, the amount of stimuli they could handle and the following consequences. We identified this as a barrier, since the outdoor environment often was perceived as being too stimulating for some residents, resulting in negative consequences, which could possibly affect their colleagues:

*”If you have too much involvement of those who are cognitively challenged, those who might subsequently get angry, then you leave the evening team with an angry resident, one who might have externalizing behaviour, because we went outside with them.”* Employee, case 03.

Thus, to be able to avoid detrimental consequences, employees felt the need to predict consequences before initiating outdoor activities.

#### Professional identity

Another barrier was the employees’ professional identity, since it affected their willingness to use the outdoor areas more. During the interviews, the employees explained that the main priority of their profession is to care for the resident and put their needs above their own. Many of the employees believed that they would benefit from being more outdoors, but since it is not necessarily the resident’s need or desire, they would not prioritize it. One employee explained this:

*“It’s the general attitude that when this is the job, then we put ourselves aside when we are at work. It’s my needs over here. It’s the resident’s need that is the main focus.”* Employee, case 01.

From the managers’ perspective, they felt that the employees should prioritize themselves and their own work environment higher. For example, one manager explained that she hoped this study would make employees more aware of what would improve their own work environment.

### Automatic motivation

#### Routines

We found that having integrated outdoor activities as routines facilitated more time outdoors. In this case, we found that automatic motivation was closely linked to the caring requirements and planning. We observed that one nursing home had a routine of planning walks with residents once a week, which they wrote into a weekly schedule. Other nursing homes did more spontaneous planning considering multiple factors (needs of residents, staffing and weather conditions) before going outdoors. This was mentioned in the interviews as well. One manager described the benefits of routine planning:

*“Simply, just write it in [the employees’] daily plan. Basic planning. Then we will do it. (…) But more systematically and clear planning of it.”* Manager, case 03.

Another manger explained, that during Covid-19 the nursing homes were ‘forced’ to create and plan more outdoor initiatives, such as outdoor concerts or food trucks, in order to make social gatherings for the residents. Going outdoors became a routine care task because it was necessary for the nursing home to do.

### Psychological capability

#### Knowledge about the residents

We found that having specific knowledge about the residents was a psychological capability that facilitated outdoor activities. During interviews, employees shared perspectives on how this knowledge could be used to persuade residents to go outdoors. Some found it useful to make the outdoor environment relevant for the residents, e.g., using outdoor experiences as a starting point for conversation about childhood memories. Others said that they used outdoor experiences as a strategy to calm or cheer up residents. In some cases, the employees even knew that some residents needed a more firm approach:

*”(…) If you ask [the residents] if they want to go for a walk, their safe answer is always no because they don’t know what to expect. So sometimes, we chose to not even ask them or to wait until the very last minute (…), and then they walk along. So, in a way they don’t get to choose.”* Employee, case 01.

#### Lack of ideas

Lack of ideas for outdoor activities seemed to be a hindering factor for going outside. In the interviews, the employees found it challenging to think of outdoor activities when asked to describe their dream scenario. The purpose of this question was to be creative and look past any possible barriers. However, many employees struggled to do so and felt retained by rules and safety.

*”(…) It becomes quite a mouthful. There are many precautions and many other things that you don’t just do. You can dream it, but making it a reality is difficult because it has something to do with safety.”* Employee, case 02.

Consequently, our interviews revealed that the employees mostly described activities that they had previously done, such as larger events or activities linked to a special occasion. Some employees explained the struggles of scaling the activity to fit as many residents as possible due to their different physical and mental stages.

One employee had a different educational background compared to the remaining staff, as she was a former childcare pedagogue. We observed, during both the interviews and field visits, that she had the psychological capability to generate many ideas for new outdoor activities as well as initiating an activity group. The remaining staff struggled to equally generate ideas as well as follow up on her ideas. In this specific context, it seemed that educational background and previous experiences might affect the capability to generate ideas for outdoor activities.

#### Taking responsibility

We found that an important facilitating factor was to allocate responsibility for initiating and conducting outdoor activities. One manager mentioned in the interview that a controlled effort is needed, otherwise these initiatives will disappear among more prioritized care tasks. One employee supported this as she explained that the responsibility mostly lies at everybody:

“*It will just require that we all took initiative and plan it (…) I just feel somebody should mention it. I don’t know. Everybody have some thoughts—everybody have some ideas, but it just requires we all at some point talk about what we could do.*” Employee, case 02.

This could mean that the individual employee must be capable of coming up with ideas, take the initiative and suggest it at gatherings and afterwards perhaps plan and conduct the outdoor activity. Thus, if the employees do not feel capable of this, implementation and completion of outdoor activities is hindered. On the other hand, if one employee feels capable and is allocated this responsibility, it seems to facilitate outdoor activities. This is supported by observations in one nursing home, where one employee was responsible for the activity-plan and therefore made sure activities, also outdoors, were planned each week.

## Discussion

In this qualitative study using multiple cases, we explored facilitators and barriers on using outdoor areas in the daily work among eldercare workers in nursing homes. We used semi-structured interviews with employees and managers as well as observations of the daily work and mapping of the outdoor environment at the participating nursing homes. Our findings show a wide range of facilitators and barriers to employees’ use of outdoor areas during their workday. Facilitators and barriers emerged from five of the six subcomponents in the behaviour-oriented COM-B model (physical opportunities, social opportunities, reflective motivation, automatic motivation, and psychological capabilities), whereas one subcomponent (physical capabilities) was uncovered. The comprehensive variation in facilitators and barriers expands our existing knowledge on the topic [21, 27, 38], and it shows that a holistic approach is needed for increasing the use of outdoor areas in nursing homes.

Scheduling of activities appears to be one of the important components of increasing the use of outdoor areas as insufficient staffing was highlighted as one of the frequent barriers. The issue with insufficient staffing is a huge and known problem within the eldercare sector and seems to increase the coming years with the increasing number of elderly. However, the work force of eldercare workers do not seem to follow this trend [8]. The responsibility for scheduling outdoor activities can be placed with certain employees, so that the activities are not forgotten. Furthermore, when employees are planning the working day and coordinating the work tasks, they should schedule the outdoor activities in the same way as other scheduled activities. Our findings suggest that employees could benefit from deciding how the residents are involved in the activities and to what extent employees should be available to colleagues during the day. This indicates the necessity for adopting a new approach in organizing and allocating work tasks, taking these aspects into account. ‘The Goldilocks Principle’ is a suggested method for designing productive work in a way that enhances employee health and wellbeing [39–42]. For instance, designing a workday to have the optimal balance of various physical activities (like sitting, standing, and movement) arranged appropriately over time. Insights from these studies can serve as inspiration for rethinking the scheduling of activities (taking place outdoor) in eldercare work.

Our findings also indicate that the eldercare workers’ professional identity hindered the use of outdoor areas as the eldercare workers put themselves and their own wellbeing second after the residents. This could lead to feeling a lacking legitimacy for being outdoors. Our findings showed that employees did not perceive outdoor activities as ‘real work’, and that they felt as failing their colleagues indoors. This finding is in line with the Swedish study, who found guilt and illegitimacy as a barrier for conducting office work outdoors [21]. Therefore, increasing the feeling of legitimacy appear as another important component of increasing the use of outdoor areas. Involving both employees and residents in the outdoor activity and having the management clearly state the value of outdoor activities, could increase the feeling of legitimacy and thus make it easier for employees to prioritize outdoor activities and to create outdoor routines and traditions.

With regard to involving the residents in outdoor activities, we were surprised that the staff often assessed that it was too stimulating for residents with cognitive challenges to be outdoors, and that an outdoor activity could subsequently lead to, e.g., anger. This does not correspond to findings from other studies, which show that staying in green outdoor environments can lead to an improved emotional state for people with dementia, such as improve mood and reduced stress, agitation, anger, apathy, and depression [43–45]. We do not know whether the staff make the assessment based on experience or expectation. Still, it points to the importance of outdoor activities, targeting the individual resident’s resources and needs in relation to the activity’s content, setting, and other participants.

We found that the employees struggled to come up with ideas for ways to increase the use of their outdoor areas. Therefore, a third possible component could be considering the influence of education programs and how they can play a significant structural role if outdoor activities in nursing homes are to be increased in general. Additionally, various actors, such as project owners, advisers, nursing home employees and managers influence whether nursing homes succeed in bringing parts of the workday outdoors. A Ph.D. study conducted at a Danish nursing home found that employing action research methods were beneficial in exploring opportunities to incorporate nature into nursing home life [46]. This involved engaging both employees and residents in reflection meetings and the development of “dream scenarios”. Hence, future research could employ similar approaches to stimulate idea generation and enhance the perception of legitimacy in encouraging activities taking place outside.

In the scientific literature, knowledge about work environment related use of outdoor areas within eldercare is limited. One Australian qualitative study found that access to outdoor areas was a main contributor for eldercare workers to feel less stressed at work [47]. Their findings revealed that the eldercare workers used outdoor areas and nature for multiple purposes, including as a space for a quiet retreat/refuge and general wellbeing. This supports the findings of our study since the employees felt it could be beneficial for their own wellbeing to take breaks outdoors as it would be a break from being available. Despite the limited studies on outdoor initiatives in eldercare, there is nascent work in the field of other occupational groups. As mentioned in the introduction, outdoor related initiatives within office work has shown to contribute to a sense of wellbeing, recovery, better communication, and social relations [21]. This is in line with our findings, revealing that the eldercare workers experienced a better communication and relation to the residents when being outdoors.

Since eldercare workers are among the working groups with the highest sickness absence in Denmark [4] future studies should focus on initiatives increasing the recruitment and retention as well as reducing the sickness absence. Initiatives involving nature and outdoor environments has shown to play an important role in both mental health prevention challenges and for re-establishing mental balance after e.g. severe stress [14, 18], and even a 10-minute ‘outdoor booster break’ has shown to reduce stress significantly [23]. Thus, there is a potential for future studies to focus on increased use of outdoor areas as preventive strategies for reducing sickness absence among eldercare workers. Furthermore, future research should explore concrete initiatives that are feasible for the workplaces/nursing homes as well as the possible health benefits of an increased use of outdoor areas. As ours and other studies finds a variety of factors that can be experienced as either facilitators or barriers using the outdoor areas, and as the factors can differ from workplace to workplace, studies focusing on participatory co-creation of new forms of outdoor work may be beneficial. Finally, as our findings show that the employees have a far greater focus on the residents’ needs than their own, it may be relevant to draw on existing research that investigates how nursing home residents get outdoors more [48, 49].

The main strength of our study was the triangulation in methods (interviews, observations, and mapping of outdoor environment) and in-depth perspectives obtained from field visits in the nursing homes. Additionally, the use of the COM-B model ensured a good and transparent theoretical point of view, and it has been used in several studies to precisely illuminate facilitators and barriers to behaviour change. However, when employing the COM-B model, we narrow our attention to certain elements within the model during our data collection and analysis. This could result in us missing out on intriguing viewpoints that exist beyond this particular theoretical framework. Yet, this applies for all frameworks. Another limitation was the limited number of cases, which might influence the degree to which the knowledge gained from this study can be directly generalized to other nursing homes. The employees participating in the interviews were selected by the mangers and depended on the work schedule. This could exclude perspectives from more busy employee. However all the participating employees described the high workload in their occupation, which lead us to believe that the selection process did not affect the outcome. Another aspect is, that our cases are located in a relatively rural area in Denmark, and there may be other facilitators and barriers in nursing homes in urban areas. Thus, the external validity of our findings may be limited. However, Flyvbjerg et al. [50] points that it is possible to generalize from a relatively small number of cases, and we did experience a saturation in data during data analysis. In this perspective, our findings might be viewed as a theory of mechanisms that can be transferable to other nursing homes. At last, it was a limitation that the data collection took place in a short and specific time period (early spring) as seasons might influence the participants perspective on being outdoors. Further, the short timeframe only allowed for data collection of observations of one day, which may not have been representative of usual practice.

## Conclusion

In this qualitative study, we explored perspectives on using outdoor areas in the daily work among eldercare workers in order to improve their physical and mental health. The eldercare workers and their managers primarily expressed facilities, traditions or repetitive events, positive experiences with residents (star moments) and knowledge about the residents as facilitators for outdoor use. Frequently mentioned barriers were insufficient staffing, hierarchy in the work tasks, professional identity, and lack of ideas. These facilitators and barriers should be taken into account when designing initiatives for increased use of outdoor areas or activities of eldercare workers.

### Electronic supplementary material

Below is the link to the electronic supplementary material.

Additional file 1. File format: .pdf. Title: *Nursing home interview guide—managers.* Description of file: This file contains the interview guide used for the interviews with nursing home managers

Additional file 2. File format: .pdf. Title: *Nursing home focus group interview guide—employees.* Description of file: This file contains the interview guide used for conducting focus group interviews with nursing home employees

## Data Availability

The datasets used and/or analysed during the current study are available from the corresponding author on reasonable request.

## List of abbreviations

BCW: The Behaviour Change Wheel
COM-B model: Capability, Opportunity, Motivation—Behaviour model
