# Supplementary material for: Facilitators and barriers for using outdoor areas in the primary work tasks of eldercare workers in nursing homes
Source: BMC Health Serv Res. 2023 Nov 24;23:1300. doi: 10.1186/s12913-023-10308-x (PMC10675903; doi:10.1186/s12913-023-10308-x)
Supplement: Supplementary file 1 — Additional file 1. File format: .pdf. Title: Nursing home interview guide—managers. Description of file: This file contains the interview guide used for the interviews with nursing home managers [file 12913_2023_10308_MOESM1_ESM.docx]

# Nursing home interview guide – managers

| **Theme** | **Questions** |
| --- | --- |
| **Briefing** | My name is [NAME], and I work at the National Research Centre for the Working Environment.  First, I would like to thank you for participating in the interview today.  As a start, I will provide some information about the practical aspects of the interview, and then I will explain what we will talk about, specifically.  The interview will last approximately 1 hour. Please remember that all opinions and perspectives are welcome. They are essential for us to understand your work. I will record the entire interview on a Dictaphone. Is that acceptable to you? The audio file will be securely stored. The interview will also be transcribed and stored securely, and your name will be anonymized.  START RECORDING  I have now started the recording, and I would like to reiterate, that it is okay with you that the interview is being recorded?  The interview is about your and your colleagues' daily work, specifically how you use or can use outdoor areas in your daily work. By outdoor areas, we mean all areas that are outside – it can be in the garden, on the terrace, in the forest, etc. The interview will be divided into 3 parts: 1) first, I will ask about situations when you have been outside, 2) then about your attitudes and motivations for using outdoor areas, and 3) finally, we will discuss your potential ideal scenario for using outdoor areas.  Do you have any questions before we get started?  First, I would like you to tell me your name, age, position, the department you work in, and how long you have been working here? |
| **Mapping of the current use of outdoor areas** | Can you briefly describe your role as the leader here at the nursing home and the primary purpose of your work?  Can you provide a brief description of the employees' workday for me? How does it typically unfold?  Can you describe situations in which you initiated or supported employees being outside during a workday, individually or with residents? This could also involve special events, transportation, or breaks in the courtyard.  Follow-up questions:   - What was the situation about? - Who else was involved? - What was the background/cause? - Who initiated it? Was it you, an employee, or someone else entirely? - Is this typically, how you support initiatives or activities? - What did it mean to you? Did it have the desired effect/outcome? - Did you/they follow up in any way afterward? Did you discuss it? |
| **Attitude towards and motivation for using outdoor areas** | Now I would like to hear a bit about your attitude and motivation for supporting the use of outdoor areas at the nursing home.   - What is your attitude towards the use of outdoor areas at the nursing home? - As a leader, do you have a specific focus that employees and residents use the outdoor areas? - What qualities does the outdoor area possess? What does it contribute to daily life at the nursing home? - Do the staff have the influence to use the outdoor area, either alone or with residents? - Do you believe that spending time outdoors during the day is a part of a good life for the elderly? Do you, as a leader, have a responsibility in this regard? - What motivates you to support employees in going outdoors, for example, with residents or alone? Would you like to support the use of outdoor areas more? - The municipality aims to strengthen its position as "Denmark's outdoor capital." How does this influence the way you run the nursing home? |
| **Facilitators and barriers for increased use of outdoor areas** | Now, I would like to hear how you would like to see the outdoor areas used here at the nursing home. This can involve both employees using them alone or with residents.  Let us call it a kind of "dream scenario" for the use of outdoor areas, where, for now, we set aside the barriers.  You may consider who can initiate it, where it should take place, and what it would require, such as a new work schedule, facilities, or skills.  Take a couple of minutes to think about this if you need to.  Summary (e.g., after 1.5-2 minutes):   - How would you like to see the outdoor areas used here? - What would promote making it a reality? - What would hinder/prevent you from initiating or carrying it out here at the nursing home? - What advice would you give to a fellow manager if they were to help their employees overcome the barriers you mention? - Do you feel equipped to initiate and support this scenario at the nursing home? Do you have the necessary knowledge, ideas, and tools? - What do you think employees and residents would gain if the scenario were implemented? - Do you have any concerns regarding the scenario? Could it have unintended consequences? - What would be the smallest effort that could result in the most significant change towards increased use of the outdoor areas/the dream scenario coming true?   ***NOTE TO INTERVIEWER:***  *We should pay attention to asking about the physical facilities, organization/planning, tasks, teamwork among colleagues, their own skills/knowledge, the attitude of management, residents' preferences, and conflicts related to their primary tasks.* |
| **Debriefing** | We are almost done, and I don't have any more questions for now. Thank you very much for your contribution. It was very interesting to gain insight into your perspectives on the use of outdoor areas.   - Is there anything you'd like to ask before we conclude? - May we contact you again if we have further questions?   You are also welcome to reach out to us if you have any questions or comments. |
