# Supplementary material for: Facilitators and barriers for using outdoor areas in the primary work tasks of eldercare workers in nursing homes
Source: BMC Health Serv Res. 2023 Nov 24;23:1300. doi: 10.1186/s12913-023-10308-x (PMC10675903; doi:10.1186/s12913-023-10308-x)
Supplement: Supplementary file 2 — Additional file 2. File format: .pdf. Title: Nursing home focus group interview guide—employees. Description of file: This file contains the interview guide used for conducting focus group interviews with nursing home employees [file 12913_2023_10308_MOESM2_ESM.docx]

# Nursing home focus group interview guide – employees

| **Theme** | **Questions** |
| --- | --- |
| **Briefing** | My name is [NAME], and I work at the National Research Centre for the Working Environment.  First, I would like to thank you for participating in the interview today.  As a start, I will provide some information about the practical aspects of the interview, and then I will explain what we will talk about, specifically.  The interview will last approximately 1 hour. Please remember that all opinions and perspectives are welcome. They are essential for us to understand your work. Please feel free to complement each other or let us know if you disagree and why. I will record the entire interview on a Dictaphone. Is that acceptable to you? The audio file will be securely stored. The interview will also be transcribed and stored securely, and your name will be anonymized.  START RECORDING  I have now started the recording, and I would like to reiterate, that it is okay with you that the interview is being recorded?  The interview is about your and your colleagues' daily work, specifically how you use or can use outdoor areas in your daily work. By outdoor areas, we mean all areas that are outside – it can be in the garden, on the terrace, in the forest, etc. The interview will be divided into 3 parts: 1) first, I will ask about situations when you have been outside, 2) then about your attitudes and motivations for using outdoor areas, and 3) finally, we will discuss your potential ideal scenario for using outdoor areas.  Do you have any questions before we get started?  First, I would like you to tell me your name, age, position, the department you work in, and how long you have been working here? Then I can recognize your voices in the audio file. |
| **Mapping of the current use of outdoor areas** | You will now get 1 minute to think about how a typical workday unfolds.  *After 1 minute:*   - Can you briefly describe the workday for me? Feel free to complement each other.   You will now get 1-2 minutes to write down different situations where you can remember being outside during a workday – either alone or with the residents. This could also include special events, transportation, or a break in the courtyard.  *After 2 minutes: Review of notes.*   - What was the situation about? What were you doing? - Who was present? Are they typically present? - Is there a particular type of resident who usually participates in that situation? Who does not? - Where were you? Are these typically used areas? - Who initiated it? - What did it mean to you? Did it have an effect or a benefit? |
| **Attitude towards and motivation for using outdoor areas** | Now I would like to hear a bit about your attitude and motivation for using the outdoor areas.   - What is the attitude towards the use of outdoor areas at the nursing home? This includes the attitudes of management, employees, and residents. - Do you, as staff, have a particular focus on using the outdoor areas? Alone or with the residents? - Do you, as staff, have the influence to use the outdoor areas? Alone or with the residents? - Do you believe that spending time outdoors during the day is a part of a good life for the elderly? Why? Do you have a responsibility in this regard? - What motivates you to go outside, for example, with the residents or alone? Would you like to use the outdoor areas more? |
| **Facilitators and barriers for increased use of outdoor areas** | Now you have a couple of minutes to consider and write in the scheme on the paper, how you would like to use the outdoor areas at the nursing home. This can involve being alone or with the residents.  You can see that you also need to write who can initiate it, who should be involved, where it should take place, and what it would require. This could include things like a new work schedule, facilities, or skills. We can call it a kind of 'dream scenario' for the use of the outdoor areas.  *After 5 minutes:*  Now, let us try to go through some of your scenarios. What have you written about initiation, involvement, location, and requirements? We will discuss them together along the way.   - What do the others think about the scenario? Do you agree that it would require xxx? - What would promote making this scenario a reality? - What would be the smallest effort that could result in the most significant change toward increased use of the outdoor areas/achieving the dream scenario? - What would hinder/prevent you from initiating or carrying out this scenario? - What advice would you give to a colleague if they had to overcome or work with the barriers you mention? - Do you feel adequately prepared to initiate these scenarios at the nursing home? Do you have the necessary knowledge, ideas, and tools? - What do you think you and the residents would gain if the scenario were implemented? How would it affect both you and the residents? - Do you have any concerns about the scenario? Could it have unintended consequences?   ***NOTE TO INTERVIEWER:***  *We should pay attention to asking about the physical facilities, organization/planning, tasks, teamwork among colleagues, their own skills/knowledge, the attitude of management, residents' preferences, and conflicts related to their primary tasks.* |
| **Debriefing** | We are almost done, and I do not have any more questions for now. Thank you very much for your contribution. It was very interesting to gain insight into your perspectives on the use of outdoor areas.   - Is there anything you'd like to ask before we conclude? - May we contact you again if we have further questions?   You are also welcome to reach out to us if you have any questions or comments. |
